# SIUMB recommendations on the use of ultrasound in neoplastic lesions of the gallbladder and extrahepatic biliary tract

**DOI:** 10.1007/s40477-023-00788-2

**Published:** 2023-05-05

**Authors:** Ilario de Sio, Mirko D’Onofrio, Paoletta Mirk, Michele Bertolotto, Kateryna Priadko, Cosima Schiavone, Vito Cantisani, Giovanni Iannetti, Gianfranco Vallone, Gianpaolo Vidili, The SIUMB experts committee, Buscarini Elisabetta, Calliada Fabrizio, Di Candio Giulio, Ferraioli Giovanna, Pavlica Pietro, Piscaglia Fabio, Pompili Maurizio, Rapaccini Gian Ludovico, Romano Marcello, Serra Carla, Soresi Maurizio, Gabriella Brizi, Tarantino Luciano, Fabia Attili

**Affiliations:** 1 Department of Hepatogastroenterology, Università Degli Studi Della Campania “Luigi Vanvitelli”, Naples, Italy; 2 Department of Radiology, G.B. Rossi Hospital, University of Verona, Verona, Italy; 3 Department of Radiology, Catholic University of the Sacred Heart-Fondazione Policlinico A. Gemelli, Rome, Italy; 4 Department of Radiology, University of Trieste, Ospedale di Cattinara, Trieste, Italy; 5 Unit of Internistic Ultrasound, Department of Medicine and Science of Aging, “G. d’Annunzio” University, Chieti, Italy; 6 Department of Radiological Sciences, Policlinico Umberto I, University La Sapienza, Rome, Italy; 7 Department of Internistic Ultrasound, Civil Hospital of Pescara, Pescara, Italy; 8 Department of Medicine and Health Sciences, “V. Tiberio” of the University of Molise, Molise, Italy; 9 Department of Medical, Surgical and Experimental Sciences, University of Sassari, Sassari, Italy

**Keywords:** Ultrasound, Neoplastic lesions, Extrahepatic biliary tract, Gallbladder, Contrast-enhanced ultrasound

## Abstract

Extrahepatic biliary tract and gallbladder neoplastic lesions are relatively rare and hence are often underrepresented in the general clinical recommendations for the routine use of ultrasound (US). Dictated by the necessity of updated summarized review of current literature to guide clinicians, this paper represents an updated position of the Italian Society of Ultrasound in Medicine and Biology (SIUMB) on the use of US and contrast-enhanced ultrasound (CEUS) in extrahepatic biliary tract and gallbladder neoplastic lesions such as extrahepatic cholangiocarcinoma, gallbladder adenocarcinoma, gallbladder adenomyomatosis, dense bile with polypoid-like appearance and gallbladder polyps.

## Preamble

This document represents the results of the Italian Society of Ultrasound in Medicine and Biology (SIUMB) guideline committee’s research concerning the use of conventional and contrast-enhanced ultrasound (CEUS) in neoplastic lesions of the gallbladder and extrahepatic biliary tract.

In 2016, we started collecting data from the literature (guidelines, scientific papers, and expert opinions) published over the past 10 years about the role of ultrasound (US) and CEUS in neoplastic lesions of the gallbladder and extrahepatic biliary tract. Recommendations were formulated on the basis of the analyzed data. Further, they were assessed by a panel of Italian physicians, experts in the use of ultrasound in neoplastic lesions of the gallbladder and extrahepatic biliary tract at the “Consensus” that took place in Rome, on 16 November 2021, during the last national conference.

The results of the expert committee’s work were presented to SIUMB members on 17 November 2021, and the text, including recommendations, was then approved by the SIUMB executive bureau on 20 January 2022.

This paper is the summary of the SIUMB’s position concerning the use of US and CEUS in neoplastic lesions of the gallbladder and extrahepatic biliary tract. The aim is to present recommendation to define the cases in which it is proper to apply a more sophisticated ultrasound imaging technique, such as CEUS, and when other imaging techniques need to be used.

## Motivations and methodology

The importance of ultrasound, and in particular the use of ultrasound contrast agents (UCAs), is well recognized in Italy, however a guideline document has not been developed by SIUMB. In the light of this lack, and on the strength of 2 decades’ experience using CEUS, SIUMB set up a guidelines committee.

In the first meeting, held in Rome in September 2016, the authors carried out an analysis and selection of the already published guidelines concerning the contributions of unenhanced and enhanced ultrasound to the diagnosis of neoplastic lesions of the gallbladder and extrahepatic biliary tract.

After the analysis of international and national guidelines, the second step was to evaluate the most important papers on the role of conventional and contrast-enhanced ultrasound in the management of patients with neoplastic lesions of the gallbladder and biliary tree.

To do that, we carried out a bibliographic search by entering the following terms in PubMed: “biliary tree and cancer and contrast enhanced ultrasound “and “gallbladder and neoplasm or cancer and contrast enhanced ultrasound “.

The research was limited to the period between 2016 and 2019, and led to the identification of 261 full papers for the item biliary tree cancer and 217 full papers for the item gallbladder neoplasm.

By activating filters for clinical trials, review and meta-analyses, we reduced the search result items to 76 full papers for biliary tree cancer and 45 full papers for gallbladder neoplasm.

We proceeded to filter these documents, only including: studies conducted on humans; studies in which the use of CEUS has been evaluated in terms of the identification and characterization of neoplastic lesions of the gallbladder and biliary tree, and the reporting data in terms of sensitivity/specificity or positive and negative predictive value (PPV-NPV); studies in which Sonovue (Bracco, Italy) was the only UCA employed (we have excluded data related to the use of Sonazoid and Definity, because at the moment they are not available in our country); studies in which a qualitative evaluation of contrast medium has been performed (we have excluded studies in which quantitative assessments have been made with wash in/wash out time intensity curves, with an analysis of images using software such as Photoshop, etc.); studies in which there were at least 30 patients (with at least 10 benign and 10 malignant gallbladder and biliary tree lesions); studies published in English; and studies in which the gold standard was the histological result, the computed tomography (CT) and/or magnetic resonance imaging (MRI) diagnosis, or the clinical and radiological follow-up.

Finally, 12 full papers were chosen for biliary tree cancer and 9 full papers for gallbladder cancer (including two EFSUMB guidelines dated 2011 and 2017 respectively, relating to the use of CEUS for non-hepatic use; a joint multi-society (ESGAR, EAES, EFISDS, ESGE) guidelines dated 2017 on the management and follow-up of gallbladder polyps as well as two meta-analysis articles).

In this document, the SIUMB’s guidelines committee decided to focus mainly on the US diagnostic aspects of gallbladder and biliary tree lesions, with no recommendations regarding the evaluation of tumor response after loco-regional treatment and systemic therapy.

In drafting the final document, we decided to report the conclusions of the existing literature as recommendations, and to include the experts’ opinions on all the gallbladder and biliary tree neoplastic lesions presented.

The evidence for and strength of the recommendations is generally assessed according to the Grading of Recommendations Assessment, Development, and Evaluation (GRADE) system [1].

The strength of recommendations depends on the quality of the evidence. Each recommendation is graded as strong or weak; high-quality evidence corresponded to a strong recommendation, while a lack of or uncertain evidence resulted in a weaker recommendation.

However, in the field of neoplastic lesions of the gallbladder and biliary tree current level of evidence present in the major part of the published studies is scarce with most of the available studies being retrospective and even monocentric [2–13]. Moreover, tumors of the gallbladder and extrahepatic biliary tract are rare that has a significant impact on the sample size in the considered studies. We therefore preferred to speak of a "position paper" rather than of "guidelines”.

The SIUMB experts’ committee voted on each of the statements. Each member of the committee had the ability to approve, disapprove or abstain from voting on a particular statement. A strong consensus was reached when there was agreement in > 95%, while broad consensus was achieved when > 80% of the experts agreed.

## Neoplastic lesions of the extrahepatic biliary tree

Extrahepatic biliary tracts include the right and left hepatic ducts, their confluence, the common hepatic duct, the cystic duct and the common bile duct.

The most frequent neoplastic pathology of the extrahepatic biliary tract is represented by cholangiocarcinoma, glandular neoplasia (adenocarcinoma) originating from the cells of the ductal epithelium or from the periductal glands. Cholangiocarcinomas of the extrahepatic biliary tract are clinically characterized by jaundice/cholestasis [2, 14].

Recommendation: Ultrasound examination represents the first level examination of such patients allowing clinicians to differentiate obstructive from non-obstructive forms of jaundice/ cholestasis (strong consensus).

### Cholangiocarcinoma

Extrahepatic cholangiocarcinoma includes the perihilar form (originating from the right and left hepatic ducts, the common hepatic duct and the cystic duct) and the distal form (originating from the common bile duct).

Perihilar cholangiocarcinoma recommendations:

- Perihilar cholangiocarcinoma causes dilation of the upstream intrahepatic biliary tract, with normal extrahepatic biliary tract, while the neoplastic lesion may appear of variable echogenicity, but often is visualized as isoechoic comparing to the surrounding hepatic parenchyma and therefore poorly delineated and sometimes even invisible; in such cases, the dilation of the intrahepatic biliary tract and the lack of connection of the bile ducts to the hilum allows us to hypothesize the perihilar form of cholangiocarcinoma (strong consensus);
- CEUS helps to improve the visibility of the lesion, as well as to note the dilation of the intrahepatic biliary ducts [4, 7, 8] (strong consensus);
- Perihilar cholangiocarcinoma shows a metastatic-like appearance on CEUS, characterized by constant hypoenhancement in the portal and late venous phase that allows to better delineate the limits and margins of the lesion. The behavior of the lesion in the arterial phase can be variable: rim-like peripheral hyperenhancement, complete and/or incomplete hyperenhancement and hypoenhancement [4, 7, 8] (strong consensus).

Distal cholangiocarcinoma recommendations:

- Distal cholangiocarcinoma localized at the level of the common bile duct, only rarely (in nodular forms) can become visible as an echogenic endoluminal lesion that cannot be differentiated from stones or echogenic material (dense bile-clots), but more frequently, in relation to the periductal infiltrating type growth (periductal sclerosing forms), is not detectable by ultrasound. In such cases, therefore, US allows us only to identify the dilation of the intrahepatic biliary tract of the common bile duct and of the gallbladder (strong consensus);
- The common bile duct sometimes has a filiform or abruptly interrupted appearance in the tract affected by the neoplasm and the diagnosing requires the use of additional methods (MRI—Echoendoscopy—Endoscopic retrograde cholangiopancreatography) (strong consensus);
- It is rarely possible to note an echogenic material, without posterior acoustic shadow, located within the common biliary tract, which can simulate the presence of biliary debris, stones in formation or clots. CEUS can show the nature of the obstruction by presenting an enhancement of the lesion in case of neoplasm [4, 7, 8].

### Metastases

The extrahepatic biliary tract is rarely affected by secondary tumors of metastatic type, especially those of gastrointestinal origin (i.e. colon and stomach cancers) or melanoma and lymphoma.

Recommendations:

- At US, the metastases appear as masses that interrupt the biliary tract with an upstream dilation of the biliary tract (strong consensus);
- At CEUS the metastases can present a diffuse or peripheral hyperenhancement in the arterial phase, followed by a hypoenhancement in the portal and late phase, with an image very similar to what can be observed in the primitive forms [9] (strong consensus).

## Neoplastic and non-neoplastic lesions of the gallbladder

### Non-mobile biliary sludge

When the sludge changes with the position, it can be safely classified as benign. Sometimes biliary sludge, due to its greater density which limits movements, can be mistakenly diagnosed as a polypoid lesion.

Recommendations:

- The presence of color Doppler signals in the lesion will be indicative of a neoplastic lesion (strong consensus);
- In cases where these vascular signals are not detectable, CEUS can be used to differentiate solid lesions from the presence of biliary sludge. In particular, the absence of enhancement of the polypoid-like lesion is a sign of the presence of dense bile (sludge) (100% accuracy) [15, 16] (strong consensus).

### Adenomyomatosis of the gallbladder

Adenomyomatosis is a gallbladder pathology characterized by hyperplasia of the muscular layer of the wall with glandular-like proliferation of the lining epithelium that appears intact; glandular-like proliferation determines the presence of intramucosal cysts corresponding to Rokitansky-Aschoff's sinuses (RAS). A diffuse variant and a focal variant (the one located at the fundus is called adenomyoma of the gallbladder and can present as a mass lesion) were described and are characterized by diffuse and focal thickening of the wall. Sometimes in patients with adenomyomatosis of the gallbladder, small echogenic spots with "comet tail artifact" related to the presence of parietal cholesterolosis can be observed in the Rokitansy-Ashoff sinuses.

Recommendations:

- Ultrasound represents the imaging method of choice in its identification and characterization, with an accuracy ranging from 91.5 to 94.8% (strong consensus);
- CEUS increases the sensitivity of US in identifying RASs and in documenting the continuity of the gallbladder walls. Moreover, CEUS targeted at identifying the thickening area of the gallbladder wall shows the same degree of vascularization as the adjacent wall, although an area of hyperenhancement can occur in 15% of cases (strong consensus);
- Avascular spaces representing RASs should be explored at the internal part of the thickened wall of the gallbladder;
- RASs appear avascular at all stages of the dynamic study, regardless of their content. The identification of avascular spaces in the context of the thickened gallbladder wall points on the presence of focal adenomyomatosis [17–22] (strong consensus).

### Focal pathology of the gallbladder

Polypoid lesions of the gallbladder are identified by abdominal US examination with a prevalence ranging between 0.3 and 9.5% [23]. Gallbladder polyps can be divided into pseudopolyps or true polyps. According to a recent systematic review of the literature, pseudopolyps represent 70% of all polypoid lesions [24]. Ultrasonography has a sensitivity and specificity for the diagnosis of true polyps of the gallbladder of 83.1 and 96.3% respectively, with a positive predictive value of 14.9% (7.0% for malignant polyps) and negative predictive value of 99.7% [25].

Recommendations:

- Ultrasonography is not able to distinguish between polypoid lesions of benign and malignant origin due to its low sensitivity for malignancy of polyps (strong consensus);
- The criteria used in the therapeutic clinical management of polypoid lesions of the gallbladder, identified after ultrasound screening, take into account the size of the polyp and the presence of some risk factors of malignancy (age > 50 years; presence of primary sclerosing cholangitis; Indian ethnicity, sessile polyp with thickening of the gallbladder wall > 4 mm) [23, 26] (strong consensus);
- Polyps ≥ 10 mm in size have an increased risk of malignancy and specialist evaluation should be suggested. However, if the patient has no risk factors, an annual US follow-up is suggested if the polyp is < 6 mm, every 6 months if the polyp size is between 6 and 9 mm [23] (strong consensus).

### Adenomatous polyps

Adenomatous polyps appear as echogenic structures without an acoustic shadow, adhering to the wall and protruding into the lumen of the gallbladder.

Recommendations:

- Adenomatous polyps are either pedunculated or with a large implant base (sessile) with possible presence of a large vascular pole which is well visualized by color Doppler and especially by CEUS (strong consensus);
- Vascularization is characterized by regular vessels with a tree-like distribution. CEUS appearance is generally characterized by hyperenhancement in the arterial phase, followed by isoenhancement in the venous phase or, more rarely, by hypoenhancement. From the analysis of the literature data, there is currently no specific dynamic pattern that, following CEUS, would allow to distinguish adenoma from malignant tumor of the gallbladder [15] (strong consensus).

### Malignant neoplasia of the gallbladder

On US examination, gallbladder adenocarcinoma can appear as a solid polypoid mass protruding into the lumen; a solid mass that occupies the entire lumen of the gallbladder, often containing stones and is poorly delimited with respect to the liver parenchyma or an infiltrative form with thickened walls.

Recommendations:

- Ultrasound is not able to characterize a protruding endoluminal lesion as a malignant or benign lesion unless there are recognizable signs of extracholecystic invasion (strong consensus);
- The use of CEUS is strongly debated in this scenario and in the latest European guidelines of 2017 its use is envisaged only in the differentiation between chronic cholecystitis and neoplasia (strong consensus);
- This caution is linked to the fact that the CEUS imaging and in particular hyperenhancement in the arterial phase do not allow us to differentiate between a malignant and a benign lesion (the pattern is present in 85% of malignant tumors and in 70% of benign tumors) (strong consensus);
- According to the recent meta-analysis, the most accurate criteria in the identification and characterization of tumor pathology of the gallbladder by CEUS are represented by: (1) identification of the discontinuity of the gallbladder wall (sensitivity 82%, specificity 93%); (2) infiltration of the adjacent liver parenchyma; (3) demonstration of tortuous and irregular vessels at the level of the tumor mass with thickening of the wall (strong consensus) [27, 28].

### Addendum

During 2020–2021 other studies have been published in the field of differential diagnosis between adenomatous and cholesterol polyps [29–31], and CEUS criteria for diagnosis of malignancy of polypoid gallbladder lesions [32]. The most important conclusion of such studies are summarized here.

### Differential diagnosis between cholesterol and adenomatous polyps

The literature must be evaluated with caution as it is manly from Eastern countries, however, the most important findings in the differential diagnosis between cholesterol and adenomatous polyps are:

- Size: significantly greater mean diameter of adenomatous polyps vs cholesterol polyps (1.45–1.5 cm cut off);
- Gallbladder wall integrity: significantly more compromised wall integrity in adenomatous polyps;
- Vascular signs at color Doppler: greater vascular signals at color Doppler in adenomatous polyps;
- Mean polyp stalk diameter evaluated by CEUS: significantly larger in adenomatous polyps;
- Vascular pattern by CEUS (linear vs dotted): more frequent in adenomatous polyps.

### Differential diagnosis between benign and malignant lesions of the gallbladder

Although the literature data must be evaluated with caution as the published literature was based mainly on Eastern countries experience, the most important findings in the differential diagnosis between benign/malignant lesions of the gallbladder, are:

- Size of the lesion (larger in neoplastic lesions);
- Gallbladder wall integrity: significantly more disrupted in malignant lesions;
- The irregularity and tortuosity of the vessels at CEUS (most frequently observed in malignant lesions);
- Timing of wash out of the lesion (≤ 28 s): more frequent in malignant lesions;
- Although the studies on the wash in/out curves of the gallbladder lesion appear promising, it is believed that there is currently insufficient evidence for their use in clinical practice that raises a need for further studies [29–32].

## Data Availability

No new data were created or analyzed in this study. Data sharing is not applicable to this article.

## Abbreviations

CEUS: Contrast-enhanced ultrasound
CT: Computed tomography
EAES: European Association of Endoscopic Surgery
EFISDS: European Federation International Society for Digestive Surgery
EFSUMB: European Federation of Societies for Ultrasound in Medicine and Biology
ESGAR: European Society of Gastrointestinal and Abdominal Radiology
ESGE: European Society of Gastrointestinal Endoscopy
MRI: Magnetic resonance imaging
RAS: Rokitansky-Aschoff's sinuse
SIUMB: Italian Society of Ultrasound in Medicine and Biology
PPV: Positive predictive value
NPV: Negative predictive value
UCA: Ultrasound contrast agent
US: Ultrasound
